# Supplementary figures and images for: Influence of upper limb training and analyzed muscles on estimate of physical activity during cereal grinding using saddle quern and rotary quern
Source: PLoS One. 2021 Aug 31;16(8):e0243669. doi: 10.1371/journal.pone.0243669 (PMC8407586; doi:10.1371/journal.pone.0243669)

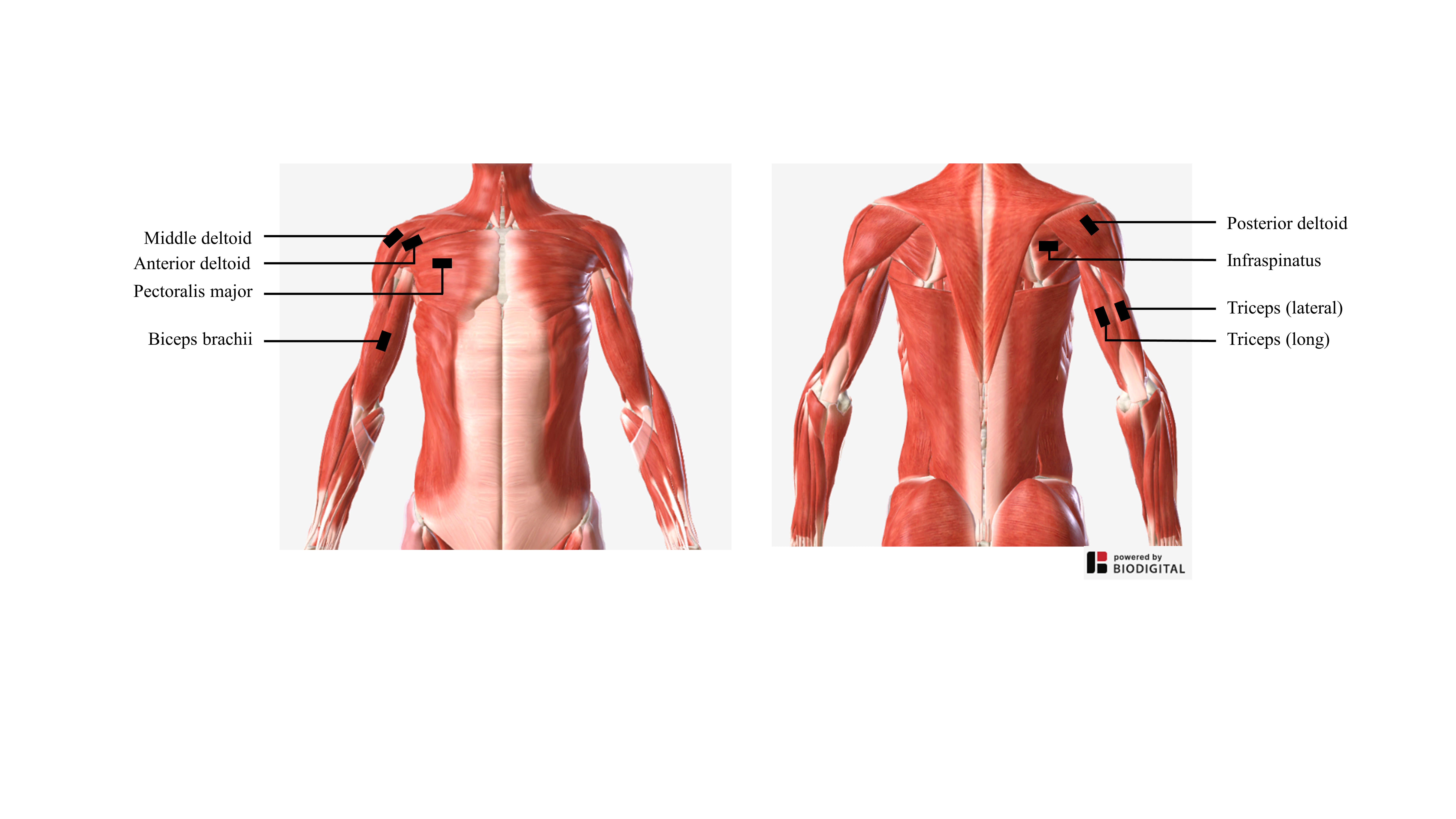

Supplement: S1 Fig — Images were generated from www.biodigital.com and edited afterwards. (TIF) [file pone.0243669.s001.tif]
