# Supplementary material for: Influence of upper limb training and analyzed muscles on estimate of physical activity during cereal grinding using saddle quern and rotary quern
Source: PLoS One. 2021 Aug 31;16(8):e0243669. doi: 10.1371/journal.pone.0243669 (PMC8407586; doi:10.1371/journal.pone.0243669)
Supplement: S1 Table — (DOCX) [file pone.0243669.s002.docx]

| **S1 Table**  Maximum voluntary contraction (MVC) values (mV) of athletes and nonathletes. | | |
| --- | --- | --- |
|  | **Athletes** | **Nonathletes** |
| **Biceps brachii** | 0.58 (0.17) | 0.43 (0.14) |
| **Anterior deltoid** | 0.63 (0.34) | 0.53 (0.18) |
| **Middle deltoid** | 0.70 (0.35) | 0.47 (0.13) |
| **Posterior deltoid** | 0.91 (0.38) | 0.55 (0.24) |
| **Infraspinatus** | 0.36 (0.15) | 0.34 (0.12) |
| **Pectoralis major** | 0.54 (0.27) | 0.28 (0.13) |
| **Triceps (lateral)** | 0.33 (0.09) | 0.35 (0.11) |
| **Triceps (long)** | 0.38 (0.16) | 0.33 (0.13) |
| Presented values are in the form of mean (standard deviation). | | |
