# Supplementary material for: Influence of upper limb training and analyzed muscles on estimate of physical activity during cereal grinding using saddle quern and rotary quern
Source: PLoS One. 2021 Aug 31;16(8):e0243669. doi: 10.1371/journal.pone.0243669 (PMC8407586; doi:10.1371/journal.pone.0243669)
Supplement: S2 Table — (DOCX) [file pone.0243669.s003.docx]

| **S2 Table**  Origin, insertion, function, and MVC tests of muscles used in the study. | | | | | |
| --- | --- | --- | --- | --- | --- |
| **Muscle** | **Origin** | **Insertion** | **Function relevant for cereal grinding** | **MVC test position** | **MVC test contraction** |
| **Biceps b.** | Supraglenoid tubercle*,* coracoid process | Radial tuberosity | Elbow flexion and supination of the forearm | Arms parallel with torso and elbow flexed 90° | Elbow flexion |
| **Anterior deltoid** | Clavicle | Deltoid tuberosity | Shoulder flexion | Shoulder flexed 90° | Shoulder flexion |
| **Middle deltoid** | Clavicle, acromion | Deltoid tuberosity | Shoulder abduction | Shoulder abducted 90° | Shoulder abduction |
| **Posterior deltoid** | Acromion, scapular spine | Deltoid tuberosity | Shoulder extension | Shoulder extended 30° | Shoulder extension |
| **Infraspinatus** | Infraspinous fossa of the scapula | Greater tubercle of the humerus | Shoulder external rotation | Arms parallel with torso and elbow flexed 90° | External rotation of humerus |
| **Pectoralis major** | Clavicle, sternum, first six ribs | Intertubercular  sulcus of humerus | Shoulder flexion, adduction, and medial rotation | Shoulders flexed 90° | Shoulder horizontal adduction |
| **Triceps b. (lateral)** | Posterior surface of humerus | Olecranon | Elbow extension | Arms parallel with torso and elbow flexed 90° | Elbow extension |
| **Triceps b. (long)** | Infraglenoid tubercle | Olecranon | Elbow extension, shoulder extension | Arms parallel with torso and elbow flexed 90° | Elbow extension |
| Muscle origin, insertion, and function are based on Gray’s anatomy [1]. Positions and contractions for MVC tests are based on Konrad [2]. | | | | | |

**S2 Table references**

1. Drake RL, Vogl AW, Mitchell AWM. Gray’s anatomy for students. 3rd ed. Philadelphia: Elsevier Churchill Livingston; 2015.

2. Konrad P. The ABC of EMG: A practical introduction to kinesiological electromyography. Scottsdale: Noraxon; 2006.
