# Supplementary material for: Influence of upper limb training and analyzed muscles on estimate of physical activity during cereal grinding using saddle quern and rotary quern
Source: PLoS One. 2021 Aug 31;16(8):e0243669. doi: 10.1371/journal.pone.0243669 (PMC8407586; doi:10.1371/journal.pone.0243669)
Supplement: S3 Table — (DOCX) [file pone.0243669.s004.docx]

| **S3 Table**  Coactivation index in athletes (lower half) and nonathletes (upper half) during saddle quern grinding. | | | | | | | | |
| --- | --- | --- | --- | --- | --- | --- | --- | --- |
|  | Biceps b. | Anterior deltoid | Middle deltoid | Posterior deltoid | Infraspinatus | Pectoralis major | Triceps b. (lateral) | Triceps b. (long) |
| Biceps b. |  | 0.70 | 0.74 | 0.54 | 0.60 | 0.58 | 0.45 | 0.30 |
| Anterior deltoid | **0.60** |  | 0.66 | 0.48 | 0.65 | 0.61 | 0.49 | 0.33 |
| Middle deltoid | **0.69** | **0.55** |  | 0.60 | 0.61 | 0.56 | 0.45 | 0.31 |
| Posterior deltoid | **0.50** | **0.43** | 0.62 |  | 0.68 | 0.62 | 0.65 | 0.53 |
| Infraspinatus | **0.57** | **0.63** | 0.67 | 0.72 |  | 0.73 | 0.67 | 0.52 |
| Pectoralis major | **0.57** | 0.76 | 0.57 | **0.49** | **0.66** |  | 0.72^a^ | 0.56 |
| Triceps b. (lateral) | **0.25** | **0.40** | **0.33** | **0.46** | **0.50** | **0.50^a^** |  | 0.71 |
| Triceps b. (long) | **0.25** | 0.35 | **0.30** | **0.48** | **0.48** | **0.46** | 0.74 |  |
| Mean; n = 10 for the athletic group and n = 25 for the nonathletic group. Bolded values in lower half denote lower coactivation index in athletes than nonathletes. All p-values > 0.1 except for coactivation of pectoralis major with triceps b. (lateral) and coactivation of biceps b. with triceps b. (lateral). P-values are the results of the Bonferroni post hoc test. See Table 1 for abbreviations of muscles.  ^a^ Difference between athletes and nonathletes has p-value = 0.048. | | | | | | | | |
