# Supplementary material for: Influence of upper limb training and analyzed muscles on estimate of physical activity during cereal grinding using saddle quern and rotary quern
Source: PLoS One. 2021 Aug 31;16(8):e0243669. doi: 10.1371/journal.pone.0243669 (PMC8407586; doi:10.1371/journal.pone.0243669)
Supplement: S4 Table — (DOCX) [file pone.0243669.s005.docx]

| **S4 Table**  Coactivation index in athletes (lower half) and nonathletes (upper half) during clockwise rotary quern grinding. | | | | | | | | |
| --- | --- | --- | --- | --- | --- | --- | --- | --- |
|  | Biceps b. | Anterior deltoid | Middle deltoid | Posterior deltoid | Infraspinatus | Pectoralis major | Triceps b. (lateral) | Triceps b. (long) |
| Biceps b. |  | 0.51 | 0.51 | 0.43 | 0.51 | 0.50 | 0.51 | 0.50 |
| Anterior deltoid | **0.42** |  | 0.49 | 0.34 | 0.57 | 0.69 | 0.52 | 0.44 |
| Middle deltoid | **0.38** | **0.43** |  | 0.72 | 0.71 | 0.33 | 0.81 | 0.71 |
| Posterior deltoid | **0.33** | **0.33** | 0.79 |  | 0.66 | 0.25 | 0.74 | 0.74 |
| Infraspinatus | **0.36** | **0.55** | **0.71** | 0.70 |  | 0.43 | 0.78 | 0.72 |
| Pectoralis major | **0.38** | **0.68** | **0.24** | **0.18** | **0.41** |  | 0.40 | 0.35 |
| Triceps b. (lateral) | **0.49** | 0.53 | **0.71** | **0.69** | **0.75** | **0.36** |  | 0.78 |
| Triceps b. (long) | **0.39** | **0.39** | **0.68** | 0.76 | **0.69** | **0.25** | **0.71** |  |
| Mean; n = 10 for the athletic group and n = 25 for the nonathletic group. Bolded values in lower half denote lower coactivation index in athletes than nonathletes. All p-values > 0.1. P-values are the results of the Bonferroni post hoc test. See Table 1 for abbreviations of muscles. | | | | | | | | |
