# Supplementary material for: Influence of upper limb training and analyzed muscles on estimate of physical activity during cereal grinding using saddle quern and rotary quern
Source: PLoS One. 2021 Aug 31;16(8):e0243669. doi: 10.1371/journal.pone.0243669 (PMC8407586; doi:10.1371/journal.pone.0243669)
Supplement: S5 Table — (DOCX) [file pone.0243669.s006.docx]

| **S5 Table**  Coactivation index in athletes (lower half) and nonathletes (upper half) during anticlockwise rotary quern grinding. | | | | | | | | |
| --- | --- | --- | --- | --- | --- | --- | --- | --- |
|  | Biceps b. | Anterior deltoid | Middle deltoid | Posterior deltoid | Infraspinatus | Pectoralis major | Triceps b. (lateral) | Triceps b. (long) |
| Biceps b. |  | 0.64 | 0.61 | 0.41 | 0.59 | 0.64 | 0.63 | 0.60 |
| Anterior deltoid | 0.67 |  | 0.50 | 0.26 | 0.49 | 0.69 | 0.51 | 0.43 |
| Middle deltoid | **0.54** | **0.48** |  | 0.61 | 0.67 | 0.43 | 0.78 | 0.61 |
| Posterior deltoid | **0.37** | 0.27 | 0.69 |  | 0.60 | 0.25 | 0.60 | 0.56 |
| Infraspinatus | **0.53** | 0.55 | 0.68 | **0.57** |  | 0.40 | 0.70 | 0.61 |
| Pectoralis major | **0.58** | **0.66** | **0.37** | **0.20** | **0.37** |  | 0.47 | 0.47 |
| Triceps b. (lateral) | 0.64 | 0.58 | **0.71** | **0.52** | 0.71 | **0.46** |  | 0.70 |
| Triceps b. (long) | **0.51** | **0.40** | **0.56** | 0.56 | **0.55** | **0.36** | **0.66** |  |
| Mean; n = 10 for the athletic group and n = 25 for the nonathletic group. Bolded values in lower half denote lower coactivation index in athletes than nonathletes. All p-values > 0.1. P-values are the results of the Bonferroni post hoc test. See Table 1 for abbreviations of muscles. | | | | | | | | |
